# Effectiveness of the REvision System and Sonic Irrigation in the Removal of Root Canal Filling Material from Oval Canals: An In Vitro Study

**DOI:** 10.3390/bioengineering9060260

**Published:** 2022-06-19

**Authors:** Marc Krikor Kaloustian, Claire El Hachem, Carla Zogheib, Walid Nehme, Louis Hardan, Pamela Rached, Naji Kharouf, Youssef Haikel, Davide Mancino

**Affiliations:** 1 Department of Endodontics, Faculty of Dentistry, Saint Joseph University, Beirut 1107 2180, Lebanon; mkaloustian75@gmail.com (M.K.K.); zogheibcarla@gmail.com (C.Z.); walidnehmeendo@gmail.com (W.N.); 2 Department of Pediatric Dentistry, Faculty of Dentistry, Saint Joseph University, Beirut 1107 2180, Lebanon; claire.elhachem@gmail.com; 3 Department of Restorative Dentistry, School of Dentistry, Saint-Joseph University, Beirut 1107 2180, Lebanon; louis.hardan@usj.edu.lb; 4 Department of Biomaterials and Bioengineering, INSERM UMR_S 1121, Strasbourg University, 67000 Strasbourg, France; pamelarached98@gmail.com (P.R.); youssef.haikel@unistra.fr (Y.H.); mancino@unistra.fr (D.M.); 5 Department of Endodontics, Faculty of Dental Medicine, Strasbourg University, 67000 Strasbourg, France; 6 Pôle de Médecine et Chirurgie Bucco-Dentaire, Hôpital Civil, Hôpitaux Universitaires, 67000 Strasbourg, France

**Keywords:** retreatment procedure, filling materials removal, revision system, sonic activation, oval-shaped canal

## Abstract

This study aimed to evaluate the effectiveness of the Endostar REvision system (Poldent, Warsaw, Poland) in the removal of filling materials from oval root canals using sonic irrigation as an additional cleaning method. Thirty human-extracted mandibular premolars with oval canals were prepared using the ProTaper Universal system (Dentsply Maillefer, Ballaigues, Switzerland) up to instrument F1 (20/.07), and then filled by the continuous wave vertical compaction technique using pulp canal sealer EWT (Sybron Dental Specialties, Orange, CA, USA). The teeth were randomly divided into two groups (*n* = 15) according to the instrumentation system and the additional cleaning method, as follows: REvision (30/.08, 25/.06) with EQ-S sonic activation (Meta Biomed, Chungcheongbuk-do, Korea), REvision (30/.08, 25/.06) without additional activation. All specimens were sectioned longitudinally at 3 and 7 mm from the apex, and analyzed using digital microscopy (KEYENCE, Osaka, Japan) to measure the total area of the residual obturation materials, followed by SEM analysis. The data on the percentage of remaining filling material were analyzed by Kruskal–Wallis one-way Analysis of Variance on ranks. None of the retreatment protocols completely removed the filling material from the root canals (*p* > 0.05); the retreatment technique using sonic activation showed statistically less residual filling materials than the retreatment technique using irrigants without activation at the coronal third (*p* < 0.05), whilst no significant difference was found between both tested groups at the apical and middle thirds (*p* > 0.05). The REvision system showed promising results in the removal of filling materials from oval canals.

## 1. Introduction

Restoring the periradicular and periapical regions is the main aim of nonsurgical root canal retreatment [1]. Around 46% of endodontic treatments are nonsurgical secondary treatments [2]. In addition, the long-term success of nonsurgical endodontic retreatment relies on the complete removal of the existing filling materials, debris, organic tissues, and microorganisms through proper cleaning, reshaping, and refilling of the root canal system [3]. The removal of the filling materials from the root canal system, especially in a curved-oval canal, presents a real challenge [4,5].

Nickel–titanium (Ni–Ti) instruments are recommended with the combination of different irrigants for facilitating the removal of old filling materials [6,7,8]. Some manufacturers have even developed instrumentation systems specifically designed for filling material removal, such as Mtwo Retreatment (VDW, Munich, Germany), ProTaper Universal Retreatment (Dentsply Maillefer, Ballaigues, Switzerland), and HyFlex Remover (Coltene Micromega). Until now, there is no validated system that ensures the complete removal of filling materials, including gutta-percha and sealer, from the root canal system [9]. The authors explain this by pointing out the unpredictable root canal anatomy and its variations, and the fact that there are regions unattained by instrumentation, especially when dealing with premolars [10].

The Endostar REvision system (Poldent, Warsaw, Poland) is a newly marketed heat retreatment system consisting of three blue files, the 30/.08, 25/.06, and 20/.04, characterized by a modified S-shaped cross-section with two cutting edges. The system has undergone a heat treatment process provided by particularly advanced technology, Azure HT technology, that offers extreme flexibility and great resistance to fracture [11]. So far, the effectiveness of REvision in the removal of filling materials from oval canals has not yet been evaluated.

Moreover, it was suggested in the literature that one should use supplementary approaches to enhance the removal of filling materials, since none of the proposed systems were able to render the root canal completely free of remnants [12,13]. Passive ultrasonic irrigation [3], sonic activation [12], Self-Adjusting File (SAF) instruments (ReDent, Ra’anana, Israel) [14], and an XP-endo Finisher (FKG Dentaire, La Chaux-de-Fonds, Switzerland) [1] were recommended.

The EQ-S (Meta Biomed, Chungcheongbuk-do, Korea) sonic activation system is a cordless device with two speeds, a multidirectional movement, and tips in three different sizes (15/.02, 25/.02, and 35/.02) that can be used at 13000 and 8000 cycles per minute, producing a 133/217 Hz frequency [14]. This device demonstrated higher smear layer removal than other commercial devices, especially at the apical third [15].

All studies have agreed that predictable removal of all the materials from the root canal system is impossible. The use of sonic activation in retreatment was recommended by some authors [16], while others did not find sonic activation useful in filling material removal [12]. The combined use of a retreatment system and sonic activation did not render the canal free of residual materials [5]. Therefore, researchers continue to find more effective techniques, instruments, and devices to promote the complete removal of root canal filling materials [9,17]. The combined use of sonic irrigation and the REvision system is a novel methodology in filling material removal.

The objective of this study was to evaluate the effectiveness of a novel Ni–Ti system, the REvision sequence, in removing filling materials from oval canals with and without the use of the EQ-S sonic irrigation. The null hypothesis was that there is no difference in the effectiveness of the REvision system in filling material removal with or without the additional usage of the EQ-S device.

## 2. Materials and Methods

### 2.1. Sample Selection

After approval by the institutional ethics committee of Saint Joseph University, Beirut, Lebanon (USJ-2017-55), 85 lower premolars, extracted for reasons unrelated to the study, were cleaned using an ultrasonic insert (1S, Satelec Acteon Group, Mérignac, France) and stored in 0.1% formocresol. Teeth were inspected under an operating microscope (Zeiss Extaro 300, Oberkochen, Germany) at x25 magnification to eliminate teeth with cracks or advanced external resorption. Mesiodistal and vestibulolingual X-rays were taken (Sopix, Satelec Acteon Group, Merignac, France) to discard teeth with treated canals, pulpal calcification, or internal resorption. Cone beam computed tomography (Newtom VGI, Verona, Italy) (CBCT) was performed, and only teeth with mature apices and a single oval canal with a moderate curvature of 15 to 22 degrees according to the Schneider technique, were included in the study [18,19,20]. Finally, 30 mandibular premolars were selected. Access cavity was prepared using an 856 diamond bur (Komet Italia SRL, Milan, Italy) with a high-speed handpiece under running water under an operating microscope, and a size #10 k-file (Dentsply Sirona, Ballaigues, Switzerland) was introduced to verify patency. This study followed the CRIS guidelines for in vitro studies, as discussed in the 2014 concept note [21].

### 2.2. Root Canal Initial Shaping and Filling

The crowns of the teeth were sectioned with a diamond disc (Kerr Dental, Bioggio, Switzerland) to standardize the root length at 15 mm. A size #15 K-file (Dentsply Sirona, Ballaigues, Switzerland) was inserted to establish the working length (WL) by reducing 1 mm from the apical foramen, and it was verified with a digital radiograph (Sopix, Satelec Acteon Group, Merignac, France). All the canals were prepared with the ProTaper Gold (Dentsply Sirona, Ballaigues, Switzerland). After this, glide path Proglider Sx, S1, S2, and F1 files were manipulated in an in/out and brushing motion with an amplitude of 3 mm to the WL, according to the manufacturer’s instructions. To preserve apical patency, a size #10 K-file was introduced after each file. Subsequently, 3 mL of 6% sodium hypochlorite (NaOCl) was flushed using a 30-gauge NaviTip needle (Ultradent, South Jordan, UT, USA) to irrigate each canal. The smear layer was dissolved with 3 mL of 17% EDTA, followed by a final rinse with 5 mL of distilled water and 3 mL of 6% sodium hypochlorite. A new sequence was used to shape each canal. At the end of the shaping procedure, sterile size F1 absorbent points (Dentsply Sirona, Ballaigues, Switzerland) were used to dry the canals, which were filled with F1 gutta-percha (GP) points (Dentsply Sirona, Ballaigues, Switzerland) and pulp canal sealer EWT (Sybron Dental Specialties, Orange, CA, USA), using the continuous wave vertical compaction technique with a fine (F) 06 plugger (Sybron Dental Specialties, Orange, CA, USA). Using the Obtura II with a 23 G tip (Obtura Spartan Endodontics, Algonquin, IL, USA), GP was injected into the canal orifice. A buccolingual and a distomesial digital radiograph was taken to validate the quality of the filling in terms of length and density. None of the teeth exhibited a poor quality of obturation; therefore, none were discarded. The access cavities were sealed with a temporary restoration material (Cavit, 3M ESPE, Seefeld, Germany). Teeth were then incubated at 37 °C for 14 days with full saturated humidity to allow the final setting [22].

### 2.3. Nonsurgical Root Canal Secondary Treatment

**Figure 1:**
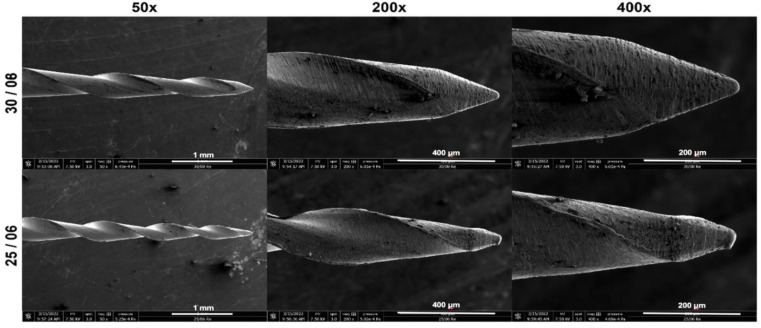
Scanning electron microscopy images demonstrating the Endostar REvision instrument (30/08 and 25/06).

After removing the temporary material using a round 856 diamond bur (Komet Italia SRL, Milan, Italy), the teeth were retreated using the REvision heat retreatment sequence. At the coronal third, 30/.08 instrument (Figure 1) was used; then, the 25/.06 instrument (Figure 1) was used at the middle and apical thirds. The files were manipulated using an Optimum Torque Reverse motor (OTR, patented by J. Morita Corp., Tokyo, Japan). The samples were then randomly divided using an online software at www.randomizer.org (accessed on 17 May 2022) to obtain two equal and balanced groups (*n* = 15) according to the following irrigation protocol:

Group 1: 12 mL of 6% NaOCl with a 30-G NaviTip needle was used and the canals were dried with F2 Paper points (Dentsply Sirona). Afterward, 3 mL of 17% EDTA was applied inside the canal for 1 min, followed by a final wash with 3 mL of 6% NaOCl.
Group 2: 12 mL of 6% NaOCl with a 30-G NaviTip needle was used. Sonic activation was then applied using the EQ-S cordless sonic endo irrigator coupled with the 25/02 tip at 13,000 cycles/min (217 Hz) [15], with 3 mm amplitude in-and-out movements without approaching the canal walls. Subsequently, 3 mL of 6% NaOCl irrigation, followed by 20 s of activation was repeated three times at 1 mm from the WL. F2 paper points (Dentsply Sirona) were used to dry the canals, and then 3 mL of 17% EDTA was applied, followed by 1 min of activation, and a final rinse with 3 mL of 6% NaOCl.

When the filling material was no longer apparent on the instrument or the canal walls under a ×16 operating microscope, the retreatment procedure was deemed complete.

### 2.4. Sectioning and Digital Microscopy Analysis

**Figure 2:**
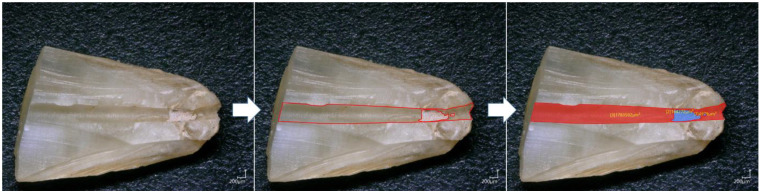
Methodology of residual materials area measurements using VHX-5000 software.

After retreatment procedures, two sections were positioned perpendicularly to the longitudinal axis of each tooth root, at 3 and 7 mm from the apex, with a diamond disc (Kerr Dental, Bioggio, Switzerland) to obtain three parts corresponding to the coronal, middle, and apical thirds. After that, to analyze the internal dentinal walls of the root canal, the specimens were sectioned by cutting two shallow longitudinal grooves (approximately 0.6 mm) in the buccolingual direction by means of a carbide bur (ref #329, KG Sorensen, São Paulo, Brazil) with a water-cooled, high-speed handpiece. The grooves were formed following the canal curvature and did not penetrate the canal. A chisel and mallet were used to split each sample. Both specimen halves were first observed using a digital microscope (KEYENCE, Osaka, Japan). One image was taken for each specimen using a 100× magnification. The micrographs at 100× magnification, showing the canal wall surface of both groups at the coronal, middle, and apical thirds, were coded for blinded analysis by an experienced examiner independent of the experiment, using the VHX-5000 communication software (KEYENCE, Osaka, Japan) to measure the total area of the residual obturation materials (gutta-percha and sealer) (Figure 2). The residual filling material percentages after retreatment were calculated by dividing the area of the residual materials measured during the analysis by the total area of the root canal of each specimen.

### 2.5. Scanning Electron Microscope Observations (SEM)

**Figure 3:**
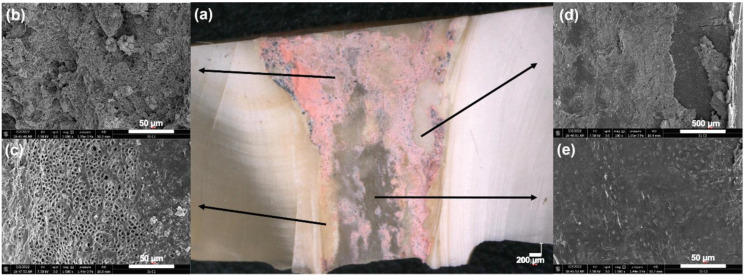
Scanning electron microscope micrographs showing the different observed colors and structures of the root canal and residual materials, which were detected under a digital microscope. (**a**) Digital microscope image; (**b**) residual materials (gutta-percha/sealer); (**c**) dentinal wall with open tubules; (**d**) residual materials–dentin interface; (**e**) dentinal walls with closed tubules.

To better distinguish dentinal walls and filling material remnants, five samples were selected from each group and further analyzed using a scanning electron microscope to verify the regions observed using the digital microscope. The specimens were dehydrated in a graded series of ethanol solutions and sputter-coated with a gold–palladium alloy (20/80 weight %) using a Hummer JR sputtering device (Technics, Rocklin, CA, USA). A Quanta 250 FEG scanning electron microscope (SEM) (FEI Company, Eindhoven, The Netherlands) with an electron acceleration voltage of 10 kV at a magnification of ×100 to ×4000 was used to analyze the prepared samples. The obtained images from SEM were considered an extra tool to help the examiner be more meticulous in measuring the total area of the residual obturation materials, which exhibit different colors under the digital microscope (Figure 3).

### 2.6. Statistical Analysis

Sigma Plot software (11.2, Systat Software, Inc., San Jose, CA, USA) was used for data analysis, with a significance level of α = 0.05. The normality of data distribution within both groups was tested using the Shapiro–Wilk test. The normality was not verified, thus, Kruskal–Wallis one-way Analysis of Variance on ranks including multiple comparison procedures (Tukey Test) was applied to determine whether significant differences existed between the different retreatment techniques for the removal of filling materials at apical, middle, and coronal thirds.

## 3. Results

The retreatment technique using activation for the endodontic irrigants showed statistically less residual filling materials than the retreatment technique using irrigants without activation at the coronal third (*p* < 0.05), whilst no significant difference was found between both tested groups at the apical and middle thirds (*p* > 0.05) (Table 1).

**Figure 4:**
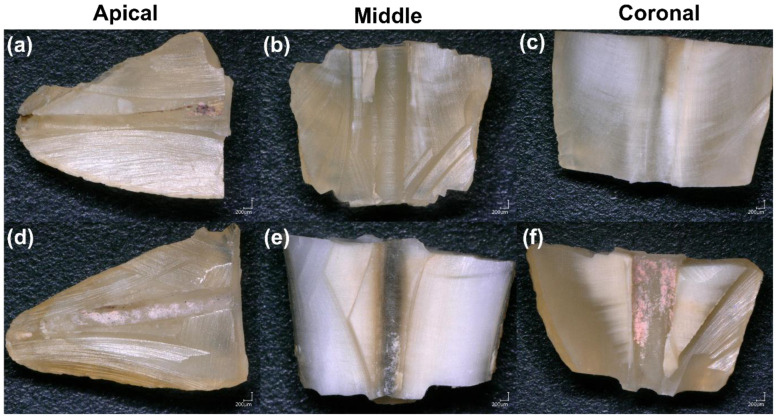
Digital microscope images demonstrate the effectiveness of the retreatment technique with irrigant activation (**a**–**c**) and without irrigant activation (**d**–**f**) in the apical, middle, and coronal thirds of the root canal.

The results of digital microscope analysis for materials removal after both final irrigation protocols are summarized in Figure 4. No statistically significant difference was found between apical–coronal and apical–middle thirds for both groups (*p* > 0.05). The middle third of each group, with and without activation, had significantly less residual obturation materials than the coronal third, (*p* = 0.021 and *p* = 0.04, respectively) (Table 1).

## 4. Discussion

Oval-shaped canals are frequently associated with insufficient preparation and cleaning during initial and secondary root canal treatment [23]. There is a discrepancy between buccolingual and mesiodistal dimensions resulting in untouched recessed areas that harbor residues of filling material, bacteria, and debris, which increases the risk of persistent infection [24,25]. Incomplete removal of filling material may hinder the prognosis of root canal secondary treatment [10]. This study aimed to determine the effectiveness of the REvision retreatment system in the removal of filling materials from oval canals with and without sonic irrigation (SI) activation as an additional cleaning method.

The results show that the REvision system alone, and coupled with SI, failed to remove 100% of the filling material from the root canals, corroborating the findings of previous studies [17,26]. However, the REvision system showed interesting results in removing filling materials without sonic activation, with 14.02% remnants in the apical third, 8.66% in the middle third, and 19.17% in the coronal third. This may be credited to the cutting efficiency of the Endostar Azure instruments with an S-shaped section [11], and to their metallurgic properties that combine the enhanced flexibility and controlled memory of martensitic files with the stiffness and hardness of austenitic files. This may also be attributed to anatomical variations. When compared with mesial canals of mandibular molars with the presence of a filled isthmus, or lower incisors with a high degree of flatness, mandibular premolars are less flattened, which favors a greater contact area of the instrument against the canal walls and, therefore, better cleaning without the need for additional methods, making the agitation of the irrigating solutions a minor factor in obtaining improved cleaning [12]. The lack of improvement in the debris score after using a supplementary cleaning method was probably also because of the high bond strength of the pulp canal sealer EWT to root dentin [27]. In recent years, the introduction of Bioceramic sealers, such as EndoSequence BC Sealer (BC Sealer, Brasseler USA, Savannah, GA, USA) and Bio-C Sealer (Angelus, Londrína, PR, Brazil), has drastically impacted the endodontic fields [28]. These sealers offer great advantages, including biocompatibility, the ability to set in humidity, and to form a chemical bond with the tooth structure, achieving an excellent hermetic seal [29]. However, a major drawback of Bioceramic sealers is their retreatability in the case of apical periodontitis [30]. The quality of evidence is low regarding the efficiency of available instrumentation in entirely removing a Bioceramic sealer [31,32]. Very few studies have evaluated the capacity of heat-treated files such as the REvision system in Bioceramic retreatment. Al Meida et al. concluded that Reciproc Blue file (VDW, Munich, Germany) did not induce dentinal defects when removing a Bioceramic sealer. Some authors suggested the use of ultrasonics, XP Endo Finisher, and Photon-initiated photoacoustic streaming (PIPS) to raise the efficiency of sealer removal, whereas the use of sonic irrigation has not yet been evaluated in the retreatability of a Bioceramic sealer [30,33]. It would be interesting to conduct a series of in vitro and clinical randomized studies to develop a feasible and reproducible protocol for bioceramic retreatment.

The results of this study suggest that the REvision retreatment system associated with sonic irrigation using EQ-S could enhance the removal of filling materials from the coronal third compared to removal by the REvision retreatment system associated with irrigation by needles (*p* < 0.05). This result may be attributed to the root canal preparation size (F1), which allowed sufficient debris transportation coronally. Moreover, no significant difference was found between the two groups for the middle and apical thirds (*p* > 0.05). Therefore, the null hypothesis must be partially rejected. This was also observed in the study of Rodriguez et al., in which there was no significant difference in the efficiency of sonic activation in canal thirds when compared with Passive Ultrasonic Activation (PUI), and in the study of Martins et al., where the EndoActivator (Dentsply Tulsa Dental Specialties, Tulsa, OK) performed similarly for all of the root canal levels evaluated, and did not improve the removal of filling material significantly [34,35]. This was also observed in the clinical study of Grischke et al., where the performance of the EndoActivator was reasonably heterogeneous with measured values of residues all over the canal [36].

No significant difference was found between the middle and apical thirds in both groups (*p* > 0.05). The study of Park et al. confirmed that the use of EDDY (VDW, Munich, Germany) sonic activation was beneficial for removing smear layers in apical regions in retreatment cases [8]. Interestingly, in the same tested group (for both groups), a significantly higher percentage of residual materials was observed at the coronal third compared to the middle third (*p* < 0.05). This was also concluded in the study of Zuolo et al., with no significant difference among the retreatment systems in the coronal third, and a lower residual filling material volume in the middle third, and in the study of Masiero et al., where most of the residual filling material was retained in the coronal third [24,37].

The effectiveness of EQ-S sonic activation is attributed to acoustic streaming within the irrigant, generated by the oscillating tip. Such streaming fields produce hydrodynamic shear stress along the endosonic files and mainly at the tip [38,39]. Even when the tip is constrained, streaming still occurs along the whole length of the file [40]; a polyamide tip in the case of EQ-S. The effect of activation is dependent on the frequency of the instrument inside the root canal and the amplitude of the swinging instrument [41]. Thus, activation might occur at lower frequencies; for example, the EQ-S irrigator operating at 217 Hz. In the literature, sonic activation was proved to be helpful in retreatment [8,16,22]. Özyürek and Demiryüek found the EndoActivator less effective than the XP-endo Finisher, whereas Grischke et al. and Martins et al. stipulated in their studies that there was no difference between the EndoActivator and PUI [12,22,36]. Differences in root canal morphology, type of filling material, and retreatment techniques could explain the contradictory results.

Various techniques have been advocated to evaluate the residual filling materials left in the root canal after retreatment, including radiographic imaging [42], clearing techniques, sectioning, and microscopic evaluation [43,44]. Recently, micro-CT imaging with high resolution has been praised in numerous studies because it is a noninvasive technique that allows accurate quantification measurements at different stages of the treatment, the specimen thus serving as its control [1,45]. However, micro-CT usage can lead to artifacts in the reconstructed images, such as beam-hardening, complicating the interpretation of the image [46]. In this study, we opted for digital microscopy followed by SEM analysis for some samples. This methodology can provide direct topographical and morphological data on the filling materials, especially the presence of sealer on the surface of the root canal walls and in dentinal tubules [44,47]. Moreover, a numerical optical microscope with composition images taken at a magnification of ×100 could be a reliable alternative to the suboptimal micro-CT axial sections resolution [44,47,48]. The teeth were sectioned with a diamond saw, and then split into two halves without touching the canal. This method was used to avoid the alteration of our results due to the debris created during sectioning procedures. Moreover, the use of SEM allowed the identification of the gutta-percha, sealer, the dentinal walls with open or closed tubules, and the residual materials–dentin areas. This could be particularly interesting for educational purposes and clinical improvement, as it allows one to visualize unprepared areas of the canals, debris, and smear layer persistence in the dental tubules.

The limitations of this study relate to its invasive methodology, consisting of sectioning the teeth, and the reduced sample size. In addition, during the sample preparation for the optical analysis, different steps could affect the results, including the use of a diamond disc and the preparation of grooves, which could generate some supplementary debris. Further in vitro study using other activation techniques and devices, such as ultrasonic and mechanical activation, should be performed. Further in vivo studies are needed to confirm the results regarding the effectiveness of the REvision system in filling material removal from oval canals with or without the additional usage of sonic irrigation.

## 5. Conclusions

The results of this study suggest that the REvision retreatment system associated with sonic irrigation using EQ-S could enhance the removal of filling materials from the coronal third compared to removal by the REvision retreatment system associated with irrigation by needles. No statistically significant difference was found between the middle and apical thirds in both groups. None of the techniques removed the root canal filling materials entirely from the oval canal of mandibular premolars. The combination of EQ-S sonic irrigation and REvision retreatment system seemed to increase the removal of filling material from the coronal third, whilst no significant difference was observed for the middle and apical thirds. Moreover, when no additional cleaning method was applied, the REvision system alone showed interesting results that merit further investigations. Additional studies could eventually evaluate the use of extended irrigation time or the use of other irrigation techniques in different anatomical situations to attain a safe and reliable removal of old filling materials from the root canal system.

## Figures and Tables

**Table 1:** Residual material percentages after both retreatment techniques with or without activation of endodontic irrigants. Different superscripted letters indicate significant differences between the different groups (*p* < 0.05).

	Apical	Middle	Coronal	Statistical Analysis (*p* < 0.05)
Activation (%)	9.59 ± 12.40	4.57 ± 8.56 ^a^	8.939.05 ^a^	*p* = 0.021
Without activation (%)	14.02 ± 20.14	8.66 ± 13.71 ^b^	19.17 ± 22.60 ^b^	*p* = 0.0040
Statistical analysis (*p* < 0.05)	No (*p* = 0.253)	No (*p* = 0.386)	Yes (*p* = 0.036)	

## Data Availability

Not applicable.

## References

[1] Crozeta B.M., Silva-Sousa Y.T.C., Leoni G.B., Mazzi-Chaves J.F., Fantinato T., Baratto-Filho F., Sousa-Neto M.D. (2016). Micro–Computed Tomography Study of Filling Material Removal from Oval-shaped Canals by Using Rotary, Reciprocating, and Adaptive Motion Systems. J. Endod..

[2] Lin S., Sabbah W., Sedgley C.M., Whitten B. (2015). A survey for endodontists in today’s economy: Exploring the current state of endodontics as a profession and the relationship between endodontists and their referral base. J. Endod..

[3] Crozeta B.M., Chaves de Souza L., Correa Silva-Sousa Y.T., Sousa-Neto M.D., Jaramillo D.E., Silva R.M. (2020). Evaluation of Passive Ultrasonic Irrigation and GentleWave System as Adjuvants in Endodontic Retreatment. J. Endod..

[4] Kfir A., Tsesis I., Yakirevich E., Matalon S., Abramovitz I. (2012). The efficacy of five techniques for removing root filling material: Microscopic versus radiographic evaluation. Int. Endod. J..

[5] Jiang S., Zou T., Li D., Chang J.W.W., Huang X., Zhang C. (2016). Effectiveness of Sonic, Ultrasonic, and Photon-Induced Photoacoustic Streaming Activation of NaOCl on Filling Material Removal Following Retreatment in Oval Canal Anatomy. Photomed. Laser Surg..

[6] Pirani C., Pelliccioni G.A., Marchionni S., Montebugnoli L., Piana G., Prati C. (2009). Effectiveness of three different retreatment techniques in canals filled with compacted gutta-percha or Thermafil: A scanning electron microscope study. J. Endod..

[7] Mollo A., Botti G., Prinicipi Goldoni N., Randellini E., Paragliola R., Chazine M., Ounsi H.F., Grandini S. (2012). Efficacy of two Ni-Ti systems and hand files for removing gutta-percha from root canals. Int. Endod. J..

[8] Park S.Y., Kang M.K., Choi H.W., Shon W.-J. (2020). Comparative Analysis of Root Canal Filling Debris and Smear Layer Removal Efficacy Using Various Root Canal Activation Systems during Endodontic Retreatment. Medicina.

[9] Duncan H.F., Chong B.S. (2008). Removal of root filling materials. Endod. Top..

[10] Gorni F.G.M., Gagliani M.M. (2004). The outcome of endodontic retreatment: A 2-yr follow-up. J. Endod..

[11] Rebeiz J., Claire E.H., El Osta N., Habib M., Rebeiz T., Zogheib C., Kaloustian M. (2021). Shaping ability of a new heat-treated NiTi system in continuous rotation or reciprocation in artificial curved canals. Odontology.

[12] Martins M.P., Duarte M.A.H., Cavenago B.C., Kato A.S., da Silveira Bueno C.E. (2017). Effectiveness of the ProTaper Next and Reciproc Systems in Removing Root Canal Filling Material with Sonic or Ultrasonic Irrigation: A Micro-computed Tomographic Study. J. Endod..

[13] Bago I., Suk M., Katić M., Gabrić D., Anić I. (2019). Comparison of the effectiveness of various rotary and reciprocating systems with different surface treatments to remove gutta-percha and an epoxy resin-based sealer from straight root canals. Int. Endod. J..

[14] Solomonov M., Paqué F., Kaya S., Adigüzel O., Kfir A., Yiğit-Özer S. (2012). Self-adjusting files in retreatment: A high-resolution micro-computed tomography study. J. Endod..

[15] Kharouf N., Pedullà E., La Rosa G.R.M., Bukiet F., Sauro S., Haikel Y., Mancino D. (2020). In Vitro Evaluation of Different Irrigation Protocols on Intracanal Smear Layer Removal in Teeth with or without Pre-Endodontic Proximal Wall Restoration. J. Clin. Med..

[16] Kaloustian M.K., Nehme W., El Hachem C., Zogheib C., Ghosn N., Mallet J.P., Diemer F., Naaman A. (2019). Evaluation of two shaping systems and two sonic irrigation devices in removing root canal filling material from distal roots of mandibular molars assessed by micro CT. Int. Endod. J..

[17] Taşdemir T., Er K., Yildirim T., Celik D. (2008). Efficacy of three rotary NiTi instruments in removing gutta-percha from root canals. Int. Endod. J..

[18] Schneider S.W. (1971). A comparison of canal preparations in straight and curved root canals. Oral Surg. Oral Med. Oral Pathol..

[19] Schirrmeister J.F., Wrbas K.-T., Meyer K.M., Altenburger M.J., Hellwig E. (2006). Efficacy of different rotary instruments for gutta-percha removal in root canal retreatment. J. Endod..

[20] de Oliveira D.P., Barbizam J.V.B., Trope M., Teixeira F.B. (2006). Comparison between gutta-percha and resilon removal using two different techniques in endodontic retreatment. J. Endod..

[21] Krithikadatta J., Gopikrishna V., Datta M. (2014). CRIS Guidelines (Checklist for Reporting In-vitro Studies): A concept note on the need for standardized guidelines for improving quality and transparency in reporting in-vitro studies in experimental dental research. J. Conserv. Dent. JCD.

[22] Özyürek T., Demiryürek E.Ö. (2016). Comparison of the Effectiveness of Different Techniques for Supportive Removal of Root Canal Filling Material. Eur. Endod. J..

[23] Ricucci D., Siqueira J.F. (2010). Fate of the tissue in lateral canals and apical ramifications in response to pathologic conditions and treatment procedures. J. Endod..

[24] Masiero A.V., Barletta F.B. (2005). Effectiveness of different techniques for removing gutta-percha during retreatment. Int. Endod. J..

[25] Vieira A.R., Siqueira J.F., Ricucci D., Lopes W.S.P. (2012). Dentinal tubule infection as the cause of recurrent disease and late endodontic treatment failure: A case report. J. Endod..

[26] Bernardes R.A., Duarte M.a.H., Vivan R.R., Alcalde M.P., Vasconcelos B.C., Bramante C.M. (2016). Comparison of three retreatment techniques with ultrasonic activation in flattened canals using micro-computed tomography and scanning electron microscopy. Int. Endod. J..

[27] da Silva Machado A.P., Câncio Couto de Souza A.C., Lima Gonçalves T., Franco Marques A.A., da Fonseca Roberti Garcia L., Antunes Bortoluzzi E., Acris de Carvalho F.M. (2021). Does the ultrasonic activation of sealer hinder the root canal retreatment?. Clin. Oral Investig..

[28] Raura N., Garg A., Arora A., Roma M. (2020). Nanoparticle technology and its implications in endodontics: A review. Biomater. Res..

[29] Camilleri J., Atmeh A., Li X., Meschi N. (2022). Present status and future directions: Hydraulic materials for endodontic use. Int. Endod. J..

[30] Zhekov K.I., Stefanova V.P. (2020). Retreatability of Bioceramic Endodontic Sealers: A Review. Folia Med..

[31] Hess D., Solomon E., Spears R., He J. (2011). Retreatability of a bioceramic root canal sealing material. J. Endod..

[32] Arul B., Varghese A., Mishra A., Elango S., Padmanaban S., Natanasabapathy V. (2021). Retrievability of bioceramic-based sealers in comparison with epoxy resin-based sealer assessed using microcomputed tomography: A systematic review of laboratory-based studies. J. Conserv. Dent. JCD.

[33] Sinsareekul C., Hiran-us S. (2022). Comparison of the efficacy of three different supplementary cleaning protocols in root-filled teeth with a bioceramic sealer after retreatment—a micro-computed tomographic study. Clin. Oral Investig..

[34] Rodrigues C.T., Duarte M.A.H., Guimarães B.M., Vivan R.R., Bernardineli N. (2017). Comparison of two methods of irrigant agitation in the removal of residual filling material in retreatment. Braz. Oral Res..

[35] Machado A.G., Guilherme B.P.S., Provenzano J.C., Marceliano-Alves M.F., Gonçalves L.S., Siqueira J.F., Neves M.A.S. (2019). Effects of preparation with the Self-Adjusting File, TRUShape and XP-endo Shaper systems, and a supplementary step with XP-endo Finisher R on filling material removal during retreatment of mandibular molar canals. Int. Endod. J..

[36] Grischke J., Müller-Heine A., Hülsmann M. (2014). The effect of four different irrigation systems in the removal of a root canal sealer. Clin. Oral Investig..

[37] de Siqueira Zuolo A., Zuolo M.L., da Silveira Bueno C.E., Chu R., Cunha R.S. (2016). Evaluation of the Efficacy of TRUShape and Reciproc File Systems in the Removal of Root Filling Material: An Ex Vivo Micro–Computed Tomographic Study. J. Endod..

[38] Ahmad M., Pitt Ford T.J., Crum L.A. (1987). Ultrasonic debridement of root canals: Acoustic streaming and its possible role. J. Endod..

[39] Walmsley A.D., Williams A.R. (1989). Effects of constraint on the oscillatory pattern of endosonic files. J. Endod..

[40] Lumley P.J., Walmsley A.D., Laird W.R. (1991). Streaming patterns produced around endosonic files. Int. Endod. J..

[41] Lumley P.J., Blunt L., Walmsley A.D., Marquis P.M. (1996). Analysis of the surface cut by sonic files. Endod. Dent. Traumatol..

[42] Baxter S., Schöler C., Dullin C., Hülsmann M. (2020). Sensitivity of conventional radiographs and cone-beam computed tomography in detecting the remaining root-canal filling material. J. Oral Sci..

[43] Raj P.K.T., Mudrakola D.P., Baby D., Govindankutty R.K., Davis D., Sasikumar T.P., Ealla K.K.R. (2018). Evaluation of Effectiveness of Two Different Endodontic Retreatment Systems in Removal of Gutta-percha: An in vitro Study. J. Contemp. Dent. Pract..

[44] Mancino D., Kharouf N., Cabiddu M., Bukiet F., Haïkel Y. (2021). Microscopic and chemical evaluation of the filling quality of five obturation techniques in oval-shaped root canals. Clin. Oral Investig..

[45] Amoroso-Silva P., Alcalde M.P., Hungaro Duarte M.A., De-Deus G., Ordinola-Zapata R., Freire L.G., Cavenago B.C., De Moraes I.G. (2017). Effect of finishing instrumentation using NiTi hand files on volume, surface area and uninstrumented surfaces in C-shaped root canal systems. Int. Endod. J..

[46] De-Deus G., Belladonna F.G., Cavalcante D.M., Simões-Carvalho M., Silva E.J.N.L., Carvalhal J.C.A., Zamolyi R.Q., Lopes R.T., Versiani M.A., Dummer P.M.H. (2021). Contrast-enhanced micro-CT to assess dental pulp tissue debridement in root canals of extracted teeth: A series of cascading experiments towards method validation. Int. Endod. J..

[47] Kharouf N., Arntz Y., Eid A., Zghal J., Sauro S., Haikel Y., Mancino D. (2020). Physicochemical and Antibacterial Properties of Novel, Premixed Calcium Silicate-Based Sealer Compared to Powder–Liquid Bioceramic Sealer. J. Clin. Med..

[48] Mancino D., Kharouf N., Hemmerlé J., Haïkel Y. (2019). Microscopic and Chemical Assessments of the Filling Ability in Oval-Shaped Root Canals Using Two Different Carrier-Based Filling Techniques. Eur. J. Dent..

